# Tonsillectomy reduces the family impact of periodic fever, aphthous stomatitis, pharyngitis and cervical adenitis (PFAPA) syndrome and improves health-related quality of life in affected children

**DOI:** 10.1186/s13023-023-02773-8

**Published:** 2023-06-20

**Authors:** Karin Rydenman, Carina Sparud-Lundin, Anna Karlsson-Bengtsson, Stefan Berg, Anders Fasth, Per Wekell

**Affiliations:** 1 Department of Pediatrics, Institute of Clinical Sciences, Sahlgrenska Academy, University of Gothenburg, Gothenburg, Sweden; 2 Department of Pediatrics, NU Hospital Group, Uddevalla, Sweden; 3 Institute of Health and Care Sciences, Sahlgrenska Academy, University of Gothenburg, Gothenburg, Sweden; 4 Department of Rheumatology and Inflammation Research, Institute of Medicine, Sahlgrenska Academy, University of Gothenburg, Gothenburg, Sweden; 5 Department of Life Sciences, Chalmers University of Technology, Gothenburg, Sweden; 6 Department of Pediatric Rheumatology and Immunology, Queen Silvia Children’s Hospital, Gothenburg, Sweden

**Keywords:** Autoinflammatory disorders, Family impact, Health-related quality of life, Periodic fever, Tonsillectomy

## Abstract

**Background:**

Periodic fever, aphthous stomatitis, pharyngitis and cervical adenitis (PFAPA) syndrome is an autoinflammatory disorder that primarily affects young children, and typically gives rise to fever episodes that recur monthly for several years. This study investigated the impact of PFAPA syndrome on the families of affected children, the health-related quality of life (HRQOL) of children with the syndrome, and how these factors were influenced by tonsillectomy.

**Methods:**

This prospective cohort study included 24 children with typical PFAPA syndrome that were referred for tonsillectomy, of whom 20 underwent the procedure. The control group consisted of randomly selected children from the general population. Family impact and HRQOL were measured using the standardized, validated questionnaires Pediatric Quality of Life Inventory™ (PedsQL™) Family Impact Module (FIM) and PedsQL™ 4.0 Generic Core Scales (GCS). Parents to children with PFAPA completed the questionnaires before and 6 months after their child underwent tonsillectomy, and HRQOL was measured both between and during PFAPA episodes. The Wilcoxon signed-rank test was used to compare data before and after tonsillectomy in the patient group, while the Mann–Whitney test was used for comparison of the patient and control groups.

**Results:**

Before tonsillectomy, children with PFAPA had significantly lower scores than the control group on the PedsQL™ FIM and the PedsQL™ 4.0 GCS during fever episodes. After tonsillectomy, all patients improved with diminished febrile episodes, which resulted in significantly higher scores regarding both family impact and HRQOL at the time of follow-up. HRQOL of in children with PFAPA improved after tonsillectomy even when compared to afebrile intervals before the procedure. The differences between PFAPA patients and controls were eliminated after tonsillectomy.

**Conclusion:**

PFAPA syndrome has a profound negative impact on the families of affected children. Tonsillectomy that leads to cessation or reduction of fever episodes eases the impact of the disease on the family. HRQOL in children with PFAPA is low during febrile episodes and similar to healthy controls in between episodes. The improvement of HRQOL in patients with PFAPA after tonsillectomy compared to the afebrile intervals before tonsillectomy highlights that the constantly recurring fevers may affect the children’s well-being even between fever episodes.

## Background

Periodic fever, aphthous stomatitis, pharyngitis and cervical adenitis (PFAPA) syndrome is an autoinflammatory disorder characterized by regularly recurring, self-limiting febrile episodes. Fever episodes are associated with one or more of the symptoms depicted in the acronym, with onset of symptoms typically occurring before 5 years of age [1]. PFAPA is considered the most common autoinflammatory disorder in children, with an annual incidence of 2.3–2.6 per 10,000 children below the age of 5 years [2, 3]. The fever episodes associated with PFAPA usually have a duration of 3–6 days and recur with intervals of 3–5 weeks. Children with PFAPA grow and develop normally and have been regarded as asymptomatic between fever episodes [1]. The recurring fever episodes usually resolve spontaneously during the childhood period, but may go on for years before this [4, 5].

Different treatment strategies are employed in children with PFAPA. Antipyretic treatments with acetaminophen and/or ibuprofen are often used during fever episodes to relieve symptoms. Single doses of corticosteroids have been shown to effectively terminate fever episodes in most patients with PFAPA, but use of corticosteroids is often associated with shortening of the fever-free intervals [6]. Tonsillectomy has been suggested to expedite recovery in most patients [7–9], but remains debated as the procedure is associated with some risks, certainty of evidence is moderate and PFAPA most often resolves spontaneously eventually [10]. Suffering from recurrent fevers for years may nevertheless have consequences for both the patients and their families. A previous study showed that the well-being of children with PFAPA is poor, with a major impact on psychosocial functioning and increased fatigue, even compared to a control group consisting of children with familial Mediterranean fever (FMF) [11]. Another study evaluated the effects of tonsillectomy on quality of life, emotional/behavioural problems and school absenteeism in children with PFAPA and found that the procedure was effective in improving these factors at the time of follow-up 3 months post-operatively [12].

In a previous qualitative study exploring parents’ experiences of PFAPA, we showed that the well-being of children with PFAPA was highly affected by symptoms during episodes and that their parents experienced increased stress, fatigue, limitations of family life and hindered professional careers [13]. The present study was constructed as a complement to that study with the intent to broaden our understanding of the measured impact of PFAPA syndrome and tonsillectomy on families of affected children, as well as the health-related quality of life (HRQOL) of children with the syndrome. Specifically, the study aims to quantify the impact of PFAPA syndrome on the families of affected children and measure the effect of tonsillectomy on this. The study also aims to investigate the HRQOL of children with PFAPA during and between fever episodes, compare this to children without the syndrome, and evaluate how HRQOL is altered by tonsillectomy.

## Materials and methods

### Study design and setting

This prospective cohort study was conducted at the Department of Pediatrics, NU Hospital Group, Uddevalla, Sweden, and the Department of Pediatric Rheumatology and Immunology, Queen Silvia Children’s Hospital, Gothenburg, Sweden, between 2016 and 2022. The study was approved by the Regional Ethics Board in Gothenburg (registration number 735-14). An amendment to the ethical permit regarding the inclusion of a control group was approved by the Swedish Ethical Review Authority (registration number 2020–00115).

### Participants

Children with typical PFAPA syndrome that were referred for tonsillectomy from the departments conducting the study and who fulfilled the inclusion and not the exclusion criteria (Table 1) were enrolled consecutively. Inclusion criteria were based on the modified Marshall criteria by Thomas et al. [1]. To ensure a homogenous study population, we added limits to the length of fever episodes and age at inclusion to these criteria, as well as exclusion criteria for atypical symptoms. Twenty-four patients were included in the study. Two of the included patients were siblings and the mother of these children answered the questionnaires for both children but 4 years apart. The control group was constructed by random selection from the Swedish population registry of children within the same age-span as the included children with PFAPA. To take non-responders into account, 60 children were initially selected for the control group. A letter was sent to their parents explaining the purpose of the study. In case of no response, we tried to reach the families by phone and sent the letter a second time. In total, 23/60 participants (38%) in the control group agreed to participate in the study and returned the questionnaires. Written informed consent was obtained from all participating parents.

**Table 1:** Inclusion and exclusion criteria for patients with PFAPA

Inclusion criteria	Exclusion criteria
Fulfil the modified Marshall criteria of PFAPA [1]: I. Regularly recurring fevers with an early onset (< 5 years of age) II. Constitutional symptoms in the absence of upper respiratory infection with at least 1 of the following clinical signs: (a) Aphthous stomatitis (b) Cervical lymphadenitis (c) Pharyngitis III. Exclusion of cyclic neutropenia IV. Completely asymptomatic between episodes V. Normal growth and development Duration of fever episodes less than 7 days Age up to 7 years at the time of inclusion	In association with fever episodes had any of the following: Skin rash Arthritis Severe abdominal pain Diarrhoea Thoracic pain Spleen enlargement Sensorineural hearing impairment Cold-induced symptoms Unable to understand written Swedish

Inclusion criteria were based on the modified Marshall criteria by Thomas et al. [1]. To ensure a homogenous study population, limits to the length of fever episodes and age at inclusion, as well as exclusion criteria for atypical symptoms, were added

*PFAPA:* Periodic fever, aphthous stomatitis, pharyngitis and cervical adenitis

### Measures

All families included in the study filled out a questionnaire containing background questions relating to their family situation and the child’s medical history, including descriptions of fever episodes in children with PFAPA. Family impact and HRQOL were measured using the standardised, validated questionnaires Pediatric Quality of Life Inventory™ (PedsQL™) Family Impact Module (FIM) and PedsQL™ 4.0 Generic Core Scales (GCS), respectively. PedsQL™ FIM gives a quantitative indicator of the family functioning as a result of their child’s health, and parent’s self-reported HRQOL. The questionnaire encompasses parents’ physical, emotional, social and cognitive function, as well as communication and worry [14]. PedsQL™ 4.0 GCS was designed to measure the core health dimensions delineated by the World Health Organization (WHO) and are composed of developmentally appropriate forms for children in different age groups. The parent proxy-reports for toddlers aged 2–4 years and young children aged 5–7 years were used in this study, allowing parents to assess their children’s physical, emotional and social functioning, as well as their functioning in day care/school [15]. The questionnaires were answered by one parent to each child and parents decided amongst themselves who should be the responder. For ages 5 and up, a child self-report form is also available. As most children in this study were below 5 years of age at the time of inclusion, the child self-report was not used. Validated Swedish translations of both questionnaires were used for this study [16, 17].

PedsQL™ FIM and PedsQL™ 4.0 GCS were completed at inclusion by all families. Parents to children with PFAPA completed two copies of the PedsQL™ 4.0 GCS at inclusion—one marked “During fever episodes” and the other marked “Between fever episodes”. This group was also asked to keep a diary of fever episodes during the study period and completed the questionnaires again 6 months after tonsillectomy. In the control group, the questionnaires were only answered on one occasion.

### Data analysis

The PedsQL™ scales were scored according to The PedsQL™ Scoring Algorithm [18], where items are reversely scored and linearly transformed to a 0–100 scale, so that higher scores indicate better outcome. The total scale score for each child was obtained by computing the mean of all items on the scale. Scores were also calculated for the pre-defined subscales (PedsQL™ FIM subscales Parent HRQOL summary score and Family functioning summary score, and PedsQL™ 4.0 GCS subscales Physical health summary score and Psychosocial health summary score). Microsoft Excel (version 2018) was used to aggregate data from the study protocols. IBM SPSS Statistics (version 28.0.0.0) was used for statistical analyses. The Wilcoxon signed-rank test was used to compare data before and after tonsillectomy in the patient group, while the Mann–Whitney test was used for comparison of the patient and control groups. *p* values of < 0.05 were considered to be statistically significant.

## Results

### Participants

Of the 24 patients with PFAPA that were referred for tonsillectomy and included in the study, 20 (83%) completed the procedure. Four patients improved spontaneously while waiting for the procedure and did not undergo surgery. These patients were therefore excluded from further analysis and did not complete the questionnaires at the second time point. Before tonsillectomy, children with PFAPA suffered a median of 5 febrile episodes (range 3–9) during a period of 6 months. The median length of fever episodes was 5 days (range 3–9 days). After tonsillectomy, all children improved, and 18/20 (90%) patients reported complete resolution of symptoms with no PFAPA episodes 6 months after tonsillectomy. The remaining two patients reported mild symptoms (periodically slightly enlarged cervical lymph nodes in one patient, one episode with elevated body temperature and headache in the other).

Children in the control group were slightly older (median age 5 years, range 3–7) than those with PFAPA (median age 4 years, range 2–7) at the time of inclusion, and the proportion of males versus females was higher in the PFAPA group (16/24 (67%) males vs 8/24 (33%) females) compared to the controls (9/23 males (39%) vs 14/23 females (61%); Table 2). In both groups, the mother was the responding parent in about 80% of cases. The number of children living at home, as well as the educational level and marital status of the responding parent were similar in both groups. Response rate in the control group was 23 out of 60 (38%).

**Table 2:** Characteristics of patients with PFAPA, controls, and their families at inclusion

	Patients with PFAPA (n = 24)	Controls (n = 23)
Child		
Age in years, median (range)	4 (2–7)	5 (3–7)
Male, n (%)	16 (67)	9 (39)
Female, n (%)	8 (33)	14 (61)
Responding parent		
Mother, n (%)	20 (83)	18 (78)
Father, n (%)	4 (17)	5 (22)
Median age in years (range)	34 (26–46)	35 (31–47)
No. of children living at home, median (range)	2 (2–4)	2 (1–4)
Highest level of education of responding parent		
Compulsory school, n (%)	0 (0)	0 (0)
Secondary school, n (%)	9 (38)	8 (35)
University, n (%)	15 (63)	15 (65)
Marital status of responding parent		
Married/cohabitants, n (%)	24 (100)	21 (91)
Divorced/separated, n (%)	0 (0)	2 (9)

### Family impact and HRQOL in PFAPA patients and controls

**Fig. 1:**
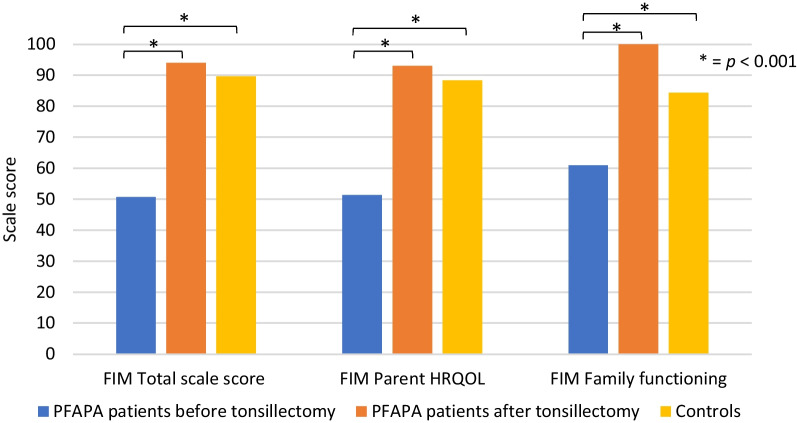
Median scores on PedsQL FIM total scale and the two subscales Parent HRQOL summary score and Family functioning summary score in PFAPA patients before vs after tonsillectomy, and controls. The Wilcoxon signed-rank test was used to compare data before and after tonsillectomy in the patient group, while the Mann–Whitney test was used for comparison of the patient and control groups. There were significant differences between PFAPA patients before and after tonsillectomy and between PFAPA patients before tonsillectomy and controls across all scales. There were no significant differences between PFAPA patients after tonsillectomy and controls

PFAPA patients showed substantially lower total scale score on the PedsQL™ FIM before as compared to after tonsillectomy (*p* < 0.001; Fig. 1). The same was seen on the two subscales Parent HRQOL summary score and Family functioning summary score. Total scale score of the FIM as well as scores on the two subscales were also significantly lower in PFAPA patients before tonsillectomy than in controls (*p* < 0.001; Fig. 1). After tonsillectomy, PFAPA patients improved to the same levels as the controls regarding the total scale score as well as the scores on the two subscales.

**Fig. 2:**
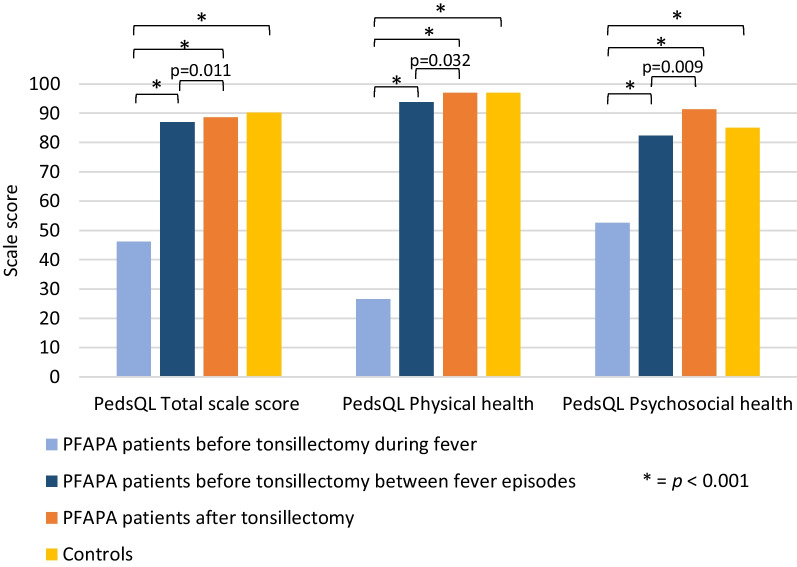
Median scores on PedsQL GCS Total scale and the two subscales (Physical health summary score and Phychosocial health summary score) in PFAPA patients during vs between fever episodes before tonsillectomy, PFAPA patients after tonsillectomy, and controls. The Wilcoxon signed-rank test was used to compare data before and after tonsillectomy in the patient group, while the Mann–Whitney test was used for comparison of the patient and control groups. Significant differences are marked in the figure. There were no significant differences between PFAPA patients between episodes before tonsillectomy and controls, nor between PFAPA patients after tonsillectomy and controls

The scores for overall health-related quality of life and physical and psychosocial functioning as measured by the PedsQL™ GCS Total scale and the subscales Physical health summary score and Psychosocial health summary score  were lower in PFAPA patients during fever episodes before tonsillectomy compared to the other groups (*p* < 0.001; Fig. 2). There was no statistically significant difference between PFAPA patients in the afebrile intervals before tonsillectomy as compared to controls. PFAPA patients did, however, show a statistically significant improvement of HRQOL on the total scale score as well as on both subscales after tonsillectomy compared to the afebrile intervals before tonsillectomy.

Taken together, all patients with PFAPA improved with diminished febrile episodes after tonsillectomy, which resulted in substantially higher scores regarding both family impact and HRQOL across all scales at the time of follow-up. The differences between PFAPA patients and controls were eliminated after tonsillectomy.

## Discussion

In this prospective cohort study, we show that PFAPA syndrome has a considerable negative impact on families of affected children that are referred for tonsillectomy. After the procedure and as the recurring fever episodes disappear, the impact of the disease ceases. The study also shows a reduction in HRQOL for children with PFAPA during febrile episodes, while the scores of PFAPA patients between febrile episodes before tonsillectomy did not differ significantly from controls. After tonsillectomy, HRQOL improved in children with PFAPA, and the scores were significantly higher than in the afebrile periods before surgery. This indicates that even though patients with PFAPA are defined as asymptomatic between episodes [1], their well-being may still be affected by the recurring fevers. It can, however, not be excluded that the improvement ensues from a higher scoring from the parents after tonsillectomy due to a sense of relief once they realize that the burdensome febrile episodes have ceased, and that the intervention has been effective. By describing HRQOL of the child and their parents, as well as functioning of the family before and after tonsillectomy, this study adds to the evidence base that is used when evaluating the risk-to-benefit balance of tonsillectomy in children with PFAPA prior to deciding if the child should undergo tonsillectomy or not.

Our study demonstrates that a periodic disease such as PFAPA in a child imposes a great burden on the entire family, including lowering of parents’ HRQOL and reduced family functioning. These findings validate the findings from a previous study by our group with another cohort of PFAPA patients [13] that employed a qualitative approach to analysing the experiences of having a child with PFAPA syndrome. Together, our studies emphasise that although the PFAPA syndrome is not life threatening, doesn’t give rise to any known long-term sequelae and eventually resolves spontaneously in most patients, having a child with constantly recurring fever episodes substantially impacts the lives of affected families.

A few studies have previously examined HRQOL in children with PFAPA and the results in our study support what has hitherto been found about how the syndrome influences affected children. Grimwood et al. showed that children with PFAPA have lower scores on PedsQL™ GCS than children with FMF and conclude that the well-being of children with PFAPA is poor [11]. Karayağmurlu et al. analysed quality of life in children with PFAPA before and after tonsillectomy and showed that the procedure was effective in improving quality of life and emotional/behavioural problems, but their study was limited by a short follow-up time of 3 months and the absence of a control group [12]. Unlike these studies, our study measured HRQOL during and between fever episodes separately in children with PFAPA. We believe that this approach is appropriate given the periodic nature of the disease and that it illuminates the effect of the febrile episodes on the children. Nonetheless, this might render the comparison of HRQOL between children with PFAPA and healthy controls, who gave a global appreciation of their general well-being, more difficult to interpret. Our study is also limited by the small sample size and the low response rate in the control group. There were some differences between the patient and the control group regarding child age and gender distribution, which might affect the results. In addition, we only included patients with PFAPA that we defined as typical, which meant that patients older than 7 years or who displayed atypical features as defined in Table 1 were excluded. While this increases the diagnostic certainty and ensures a homogenous study population, it also means that the results may not be generalizable to all patients with PFAPA.

The impact of a disease on a patient and his or her family is complicated and multi-dimensional. The PedsQL™ GCS is widely used in a broad range of paediatric patient populations as well as with healthy children [19]. The PedsQL™ FIM is constructed to be used as a complement to PedsQL™ GCS and has been shown to provide a reliable and valid measure of parent HRQOL and family functioning in children with chronic medical conditions as well as in community samples [14, 20]. While use of standardized, validated questionnaires in this study provides an advantage in the sense that the method has been previously evaluated, it also constitutes a limitation regarding the depth in which these complex issues are explored. As the scales are designed to be generic, it can be expected that they do not fully capture all aspects of each specific disease.

In our study, only one of the parents to each child answered the questionnaires and they decided amongst themselves who should be the responder. The result of this was that approximately 80% of responders in both the PFAPA group and the control group were mothers. This reflects the pattern in our clinics, where most children are accompanied by their mothers when they visit us, and probably also the situation in society at large were mothers commonly take a larger responsibility than the fathers in the care of the children. Numbers were too low to make meaningful comparisons regarding whether the impact of having a child with PFAPA differs between mothers and fathers in this study. Future studies are needed to address this issue.

Tonsillectomy is a common treatment option in children with PFAPA syndrome and has shown good results [10], but it is still somewhat controversial as the disease is self-limiting and usually resolves spontaneously after approximately 5–7 years [4]. Complications of surgery includes mild afflictions such as throat pain, post-operative nausea and vomiting, feeding difficulties and dehydration, as well as potentially severe and life-threatening events such as respiratory compromise and bleeding [21, 22]. Although the aim of this study was not to evaluate the results of tonsillectomy on the febrile episodes, our data support the effectiveness of the procedure in this selected group of patients. A shared decision-making approach has been suggested, involving the patient and caregiver in the consideration of benefits and potential harm of the procedure. This approach is routinely used in our clinics and in this study the impact of PFAPA syndrome was only measured in the subgroup of patients that were referred for tonsillectomy after such shared decision-making. Negative effects of tonsillectomy were not evaluated, and future studies are needed to further explore this and provide guidance on how to identify patients with PFAPA that benefit the most from tonsillectomy. Further studies are also needed to examine the impact of PFAPA in the whole patient group and how it develops over time in patients that do not go through tonsillectomy.

## Conclusions

PFAPA syndrome has a profound negative impact on the families of affected children. Tonsillectomy that leads to diminished fever episodes reduces the impact of the disease on the family. HRQOL in children with PFAPA is low during febrile episodes and similar to healthy controls in between episodes. The improvement of HRQOL in patients with PFAPA after tonsillectomy compared to the afebrile intervals before tonsillectomy highlights that the constantly recurring fevers may affect the children’s well-being even between fever episodes.

## Data Availability

The datasets used and/or analysed during the current study are available from the corresponding author on reasonable request.

## Abbreviations

PFAPA: Periodic fever, aphthous stomatitis, pharyngitis and cervical adenitis syndrome
HRQOL: Health-related quality of life
PedsQL™: Pediatric Quality of Life Inventory™
FIM: Family Impact Module
GCS: Generic Core Scales
FMF: Familial Mediterranean fever
WHO: World Health Organization

## References

[1] Thomas KT, Feder HM, Lawton AR, Edwards KM (1999). Periodic fever syndrome in children. J Pediatr.

[2] Forsvoll J, Kristoffersen EK, Oymar K (2013). Incidence, clinical characteristics and outcome in Norwegian children with periodic fever, aphthous stomatitis, pharyngitis and cervical adenitis syndrome; a population-based study. Acta Paediatr.

[3] Rydenman K, Fjeld H, Hätting J, Berg S, Fasth A, Wekell P (2022). Epidemiology and clinical features of PFAPA: a retrospective cohort study of 336 patients in western Sweden. Pediatr Rheumatol Online J.

[4] Wurster VM, Carlucci JG, Feder HM, Edwards KM (2011). Long-term follow-up of children with periodic fever, aphthous stomatitis, pharyngitis, and cervical adenitis syndrome. J Pediatr.

[5] Yıldız M, Haslak F, Adrovic A, Ülkersoy İ, Gücüyener N, Şahin S (2022). Periodic fever, aphthous stomatitis, pharyngitis, and adenitis syndrome: a single-center experience. Turk Arch Pediatr.

[6] Feder HM, Salazar JC (2010). A clinical review of 105 patients with PFAPA (a periodic fever syndrome). Acta Paediatr.

[7] Renko M, Salo E, Putto-Laurila A, Saxen H, Mattila PS, Luotonen J (2007). A randomized, controlled trial of tonsillectomy in periodic fever, aphthous stomatitis, pharyngitis, and adenitis syndrome. J Pediatr.

[8] Garavello W, Romagnoli M, Gaini RM (2009). Effectiveness of adenotonsillectomy in PFAPA syndrome: a randomized study. J Pediatr.

[9] Gozen ED, Yildiz M, Kara S, Tevetoglu F, Haslak F, Adrovic A (2023). Long-term efficacy of tonsillectomy/adenotonsillectomy in patients with periodic fever aphthous stomatitis pharyngitis adenitis syndrome with special emphasis on co-existence of familial Mediterranean fever. Rheumatol Int.

[10] Burton MJ, Pollard AJ, Ramsden JD, Chong LY, Venekamp RP (2019). Tonsillectomy for periodic fever, aphthous stomatitis, pharyngitis and cervical adenitis syndrome (PFAPA). Cochrane Database Syst Rev.

[11] Grimwood C, Kone-Paut I, Piram M, Rossi-Semerano L, Hentgen V (2018). Health-related quality of life in children with PFAPA syndrome. Orphanet J Rare Dis.

[12] Karayağmurlu A, Aytaç İ (2020). Pre- and postoperative quality of life and emotional/behavioural problems in children with PFAPA. Int J Pediatr Otorhinolaryngol.

[13] Sparud-Lundin C, Berg S, Fasth A, Karlsson A, Wekell P (2019). From uncertainty to gradually managing and awaiting recovery of a periodic condition—a qualitative study of parents experiences of PFAPA syndrome. BMC Pediatr.

[14] Medrano GR, Berlin KS, Hobart DW (2013). Utility of the PedsQL family impact module: assessing the psychometric properties in a community sample. Qual Life Res Int J Qual Life Asp Treat Care Rehabil.

[15] Varni JW, Seid M, Kurtin PS (2001). PedsQL 4.0: reliability and validity of the Pediatric Quality of Life Inventory version 4.0 generic core scales in healthy and patient populations. Med Care.

[16] Tiberg I, Hallström I (2009). Translation and testing of a quality of life instrument: the PEDSQL Family Impact Module [Swedish]. Nord J Nurs Res.

[17] Petersen S, Hagglof B, Stenlund H, Bergstrom E (2009). Psychometric properties of the Swedish PedsQL, pediatric quality of life inventory 4.0 generic core scales. Acta Paediatr.

[18] Scaling and Scoring of the Pediatric Quality of Life Inventory™ PedsQL™: Mapi Research Trust; [updated 12/09/2022]. Available from: https://www.pedsql.org/PedsQL-Scoring.pdf.

[19] Hullmann SE, Ryan JL, Ramsey RR, Chaney JM, Mullins LL (2011). Measures of general pediatric quality of life: Child Health Questionnaire (CHQ), DISABKIDS Chronic Generic Measure (DCGM), KINDL-R, Pediatric Quality of Life Inventory (PedsQL) 4.0 Generic Core Scales, and Quality of My Life Questionnaire (QoML). Arthritis Care Res (Hoboken).

[20] Varni JW, Sherman SA, Burwinkle TM, Dickinson PE, Dixon P (2004). The PedsQL family impact Module: preliminary reliability and validity. Health Qual Life Outcomes.

[21] De Luca CG, Pachêco-Pereira C, Aydinoz S, Bhattacharjee R, Tan HL, Kheirandish-Gozal L (2015). Adenotonsillectomy complications: a meta-analysis. Pediatrics.

[22] Mitchell RB, Archer SM, Ishman SL, Rosenfeld RM, Coles S, Finestone SA (2019). Clinical practice guideline: tonsillectomy in children (update)-executive summary. Otolaryngol Head Neck Surg.

